# Long-term declines in ADLs, IADLs, and mobility among older Medicare beneficiaries

**DOI:** 10.1186/1471-2318-11-43

**Published:** 2011-08-16

**Authors:** Fredric D Wolinsky, Suzanne E Bentler, Jason Hockenberry, Michael P Jones, Maksym Obrizan, Paula AM Weigel, Brian Kaskie, Robert B Wallace

**Affiliations:** 1 Department of Health Management and Policy, College of Public Health, the University of Iowa, Iowa City, Iowa, USA; 2 Department of Internal Medicine, Carver College of Medicine, the University of Iowa, Iowa City, Iowa, USA; 3 Department of Adult Nursing, College of Nursing, the University of Iowa, Iowa City, Iowa, USA; 4 Comprehensive Access and Delivery Evaluation and Research Center (CADRE), Iowa City Veterans Administration Medical Center, Iowa City, Iowa, USA; 5 Department of Biostatistics, College of Public Health, the University of Iowa, Iowa City, Iowa, USA; 6 Kyiv School of Economics, and Kyiv Economics Institute, Kyiv, UA; 7 Department of Epidemiology, College of Public Health, the University of Iowa, Iowa City, Iowa, USA

## Abstract

**Background:**

Most prior studies have focused on short-term (≤ 2 years) functional declines. But those studies cannot address aging effects inasmuch as all participants have aged the same amount. Therefore, the authors studied the extent of long-term functional decline in older Medicare beneficiaries who were followed for varying time lengths, and the authors also identified the risk factors associated with those declines.

**Methods:**

The analytic sample included 5,871 self- or proxy-respondents who had complete baseline and follow-up survey data that could be linked to their Medicare claims for 1993-2007. Functional status was assessed using activities of daily living (ADLs), instrumental ADLs (IADLs), and mobility limitations, with declines defined as the development of two of more new difficulties. Multiple logistic regression analysis was used to focus on the associations involving respondent status, health lifestyle, continuity of care, managed care status, health shocks, and terminal drop.

**Results:**

The average amount of time between the first and final interviews was 8.0 years. Declines were observed for 36.6% on ADL abilities, 32.3% on IADL abilities, and 30.9% on mobility abilities. Functional decline was more likely to occur when proxy-reports were used, and the effects of baseline function on decline were reduced when proxy-reports were used. Engaging in vigorous physical activity consistently and substantially protected against functional decline, whereas obesity, cigarette smoking, and alcohol consumption were only associated with mobility declines. Post-baseline hospitalizations were the most robust predictors of functional decline, exhibiting a dose-response effect such that the greater the average annual number of hospital episodes, the greater the likelihood of functional status decline. Participants whose final interview preceded their death by one year or less had substantially greater odds of functional status decline.

**Conclusions:**

Both the additive and interactive (with functional status) effects of respondent status should be taken into consideration whenever proxy-reports are used. Encouraging exercise could broadly reduce the risk of functional decline across all three outcomes, although interventions encouraging weight reduction and smoking cessation would only affect mobility declines. Reducing hospitalization and re-hospitalization rates could also broadly reduce the risk of functional decline across all three outcomes.

## Background

Promoting health and preventing functional decline in older adults is a longstanding public health policy goal in the United States [1]. At the population level, some have argued that late-life functional status and disability for older Americans may be better now than it has ever been [2], but others have counter-argued that it has not [3]. Even less is definitively known, however, about improving functional health status and preventing disability among older adults in the United States at the individual level [4,5]. This involves identification of the risk factors for whether a particular older adult experiences declining functional health.

Despite numerous reports addressing risk factors among older adults in the United States for functional decline and disability onset, a variety of limitations exist. These include heterogeneity in study design and population, outcome measurement, adjustment for initial functional status, covariates considered, omitted confounders, and attrition bias. As a result, it has been "difficult to extract a coherent, dependable list of factors from which to develop prevention strategies [5]." Two recent reports [6,7] have used innovative trajectory techniques to resolve some of the more serious methodological problems. At the same time, those methodologically sophisticated studies remain somewhat limited given their focus on short-term (two-year) effects, a single functional outcome, relatively simple adjustments for respondent status (self- vs. proxy-respondents), participation in Medicare managed care (vs. fee-for-service), health status, and insufficient data to address modifiable factors such as lifestyle, continuity of care, or hospitalization patterns, as well as other salient issues like terminal drop. Moreover, by focusing on changes occurring between fixed (i.e., two-year) periods between observations for all participants, those studies have not been able to estimate aging effects *per se*. That is, they were not able to capture the effects of differential exposure to the aging process, which requires variation in the time intervals between the two functional status assessments.

Accordingly, in this study we addressed the research question of the extent of long-term functional declines in older Medicare beneficiaries who were followed for varying time lengths, and we identified the main risk factors associated with those declines. Our study design facilitates a prospective cohort analysis of long-term declines in functional status in older Medicare beneficiaries. We used baseline (1993-1994) and biennial follow-up interview data through 2006 from the nationally representative Survey on Assets and Health Dynamics among the Oldest Old (AHEAD) in the United States that are linked to Medicare claims for calendar years 1993-2007. After adjusting for other known risk factors, we focused on the associations between long-term declines in functional status with respondent status, health lifestyle, participating in Medicare managed care, continuity of care, prior hospitalizations, and terminal drop.

We focus on these risk factors for several reasons. First, although the use of proxy-respondents is an unavoidable necessity in population-based studies of older adults either because intended participants are unable or unavailable to answer for themselves, it is essential to take respondent status into consideration in all statistical analyses [8,9]. This is especially important for longitudinal assessments of changes in reported functional status (or other health outcomes) between two or more points in time when respondent status may vary over those time points, and where adjusting for respondent status is more complex. Second, health lifestyle can be modified, and doing so is a major component of most public health interventions in the United States [1] and elsewhere.

Third, because claims-reporting requirements for Medicare managed care plans [10] are somewhat different for Part B (outpatient services) than those in fee-for-service Medicare, it has generally been recommended that studies using Medicare claims should exclude participants who participate in Medicare managed care during any part of the study's observation period [11]. This, however, limits generalizability, especially given the fact that participation in Medicare managed care during the observation period for this study has risen from 5.0% in 1993 to 16.0% in 2006 [11]. Moreover, our study only uses Medicare Part B claims to measure two variables, continuity of care and primary care physician visits between the first and last functional assessments. Furthermore, we are aware of no published studies documenting the effects of the potential reporting error associated with using the Part B claims for individuals in Medicare managed care plans. Therefore, although we include participants who have some Medicare managed care experience during the observation period, we adjust for this in the same way that we did for respondent status.

Fourth, continuity of care has not previously been considered, and not only can it be modified, it will be under the *Patient Protection and Affordable Care Act *(*PPACA*) [12] recently adopted in the United States. Fifth, although prior hospitalizations have been considered as predictors of functional decline in several previous studies [13,14] and these studies reported significant effects, they were based on smaller community studies that were only able to consider functional changes occurring over the short-term (two-years). Finally, the applicability of the terminal drop hypothesis, which states that death is preceded by a decrease in cognitive functioning prior to death [15-18], especially in the year immediately before death occurs, has not previously been considered with respect to functional status decline.

## Methods

### Study cohort and sample selection

Detailed documentation of AHEAD study measures and design can be found at http://hrsonline.isr.umich.edu and have been described elsewhere [19]. Briefly, the AHEAD is conducted by the Survey Research Center (SRC) at the University of Michigan as the United States' national, omnibus health and retirement longitudinal data source for public scientific use under contract from the National Institutes of Health, and with support from the Social Security Administration and other federal agencies. AHEAD has four major objectives: (1) to monitor transitions in physical, functional, and cognitive health status among older Americans; (2) to examine the linkage between changes in physical and cognitive health and economic status (especially dis-saving [i.e., spending down one's assets] and income flows) among older Americans; (3) to examine the relationship between changes in health status and economic status in general, and intergenerational transfers in particular among older Americans; and, (4) to evaluate how familial, economic, and programmatic resources affect institutionalization, dis-saving, and health declines among older Americans. AHEAD contains a wide array of information, including: demographics, housing stock, cognitive performance, physical and functional health, assets and income, programmatic claims, dis-saving and Medicaid eligibility, family structure, care-giving, financial transfers, and out-of-pocket costs. AHEAD respondents were identified either from household screening conducted during the 1992 multi-stage cluster sampling process for a companion study of pre-retirement aged adults, or a supplemental sample of persons 80 years old or older identified from the Centers for Medicare and Medicaid Services' (CMS) Medicare Master Enrollment File. Over-samples of 1.8 times the probability of general selection were used to increase the number of African Americans, Hispanics, and residents of the State of Florida. Thus, SRC-derived weights are used to adjust for the unequal probabilities of selection due either to the multi-stage cluster and/or the over-sampling factors. An 80.4% response rate was obtained for the baseline (1993-1994) interviews, yielding 7,447 participants who were 70 years old or older at that time. Biennial follow-up interviews were conducted through the 2010-2011 cycle, although in this study only follow-up interviews conducted through the 2006-2007 cycle are used.

Of the 7,447 original AHEAD participants, 774 did not provide consent to link their survey data to the Medicare claims, reducing the potential sample size to 6,673. Linkage errors occurred for 28 of these participants, further reducing the potential sample size to 6,645. Of these 6,645 AHEAD participants, 774 had no follow-up interviews after baseline. The main reason for this was having died before the 1995-1996 follow-up interview cycle was completed on June 15, 1996, which was the case for 82%. Thus, the analytic sample for this study consisted of 5,871 AHEAD participants (78.8% of the original sample), all of whom had at least one follow-up interview and a successful linkage of the survey data to their Medicare claims.

For each participant in the analytic sample, the final follow-up interview was their last interview before either their death or the 2006-2007 follow-up interviews, whichever came first. Selection of these final follow-up interviews was facilitated by our access to the restricted AHEAD data files that contain the actual dates for the baseline and all follow-up interviews, and the Medicare Denominator Standard Analytic File (SAF) which updates participation in managed care and vital status on a monthly basis.

### Outcome assessment

The same outcome assessments and procedures were used at all waves of data collection in the AHEAD. These assessments target the (or the then) present point in time. As a result, the comparison between the baseline and the final follow-up assessment is a meaningful indicator of long-term decline. Functional status was assessed in the AHEAD using three standard measures: limitations in activities of daily living (ADLs), instrumental ADLs (IADLs), and mobility. ADL limitation was the simple count of difficulties in performing (or inabilities to perform) five activities: getting across a room, dressing, bathing or showering, eating, and getting in or out of bed. Similarly, IADL limitation was the simple count of difficulties in performing (or inabilities to perform) five activities: using a telephone, taking medication, handling money, shopping, and preparing meals. Mobility limitation was also the simple count of difficulties in performing (or inabilities to perform) four activities: climbing up and down one flight of stairs, walking several blocks, pushing and/or pulling heavy objects, and lifting or carrying 10 pounds or more.

Declines in ADL, IADL, and mobility abilities were defined as the development of at least two new activity limitations. This level of decline in ADLs, IADLs, and mobility abilities are generally viewed in the gerontology and geriatrics literatures as personally and clinically meaningful [20,21]. Nonetheless, as an added safeguard we conducted sensitivity analyses in which we defined functional status decline as the development of at least one new activity limitation on the ADL, IADL, or mobility assessments. Because these sensitivity analyses achieved equivalent results, those results are not shown below.

### Adjusting for exposure time

The final follow-up interview was the last time the participant was interviewed before dying, or their completion of the 2006 follow-up interview, whichever came first. Thus, both the amount of time from the baseline interview to the final follow-up interview, and the reasons for that amount of time varied across participants. The amount of time ranged from a minimum of 2 years to a maximum of 12 years. To adjust for the differential amounts of exposure time between the baseline and final follow-up interview (which is directly interpretable as an aging effect) we included in all analyses the number of years (which were centered at the mean to minimize collinearity) from the baseline to the final follow-up interview. To assess the potential for non-linear exposure (aging) effects, the quadratic of the centered exposure measure was also included.

### Adjusting for respondent status

We considered respondent status at both the baseline and final follow-up interview. A set of four binary indicators was created to reflect the four possible combinations: self-respondent at both interviews, self-respondent at baseline but proxy-respondent at the final follow-up, proxy-respondent at baseline but self-respondent at the final follow-up, and proxy-respondent at both interviews. Because it was both the largest and theoretically the most logical comparison group, the self-respondent at both interviews indicator was selected as the omitted or reference category. These binary indicators capture the additive effects of respondent status.

We expect participants who were self-respondents at both interviews to be the most protected from functional decline, as they are able to independently provide their own information. We also, however, expect those who were proxy-respondents at baseline but self-respondents at the final follow-up to be equally (or even better) protected from functional decline, because most of these participants either were (a) unavailable at baseline due to external obligations (e.g., jobs) with those obligations lessening afterwards (retirement), or (b) were unable to be self-respondents at baseline due to health problems from which they subsequently recovered. In contrast, we expect those who were self-respondents at baseline but relied on proxy-respondents at the final follow-up interview to be the most likely to experience functional decline because their change in respondent status is in the reverse direction. We expect participants for whom proxy-respondents were used at both interviews to have increased risk of functional decline (relative to those who were self-respondents at both interviews, the reference category), but that this increased risk would be somewhat less than that for participants who were self-respondents at baseline but for whom proxy-respondents subsequently provided the survey information. The reason for this expectation is that the functional decline for these participants is likely to have been somewhat stabilized.

To capture the interaction effect between respondent status and baseline functional status on predicting functional status at the final follow-up interview, another set of binary markers was created. This involved multiplying each of the three non-reference respondent status indicators with the baseline functional status measure. The interaction term reflecting being a self-respondent at both interviews and baseline functional status was used as the omitted or reference category. We expect that the interaction terms involving the participants for whom proxy-respondents were used at the final follow-up interview will reduce the effect of the baseline functional status measures. The reason for this is that they are unlikely to be able to detect functional declines with as much granularity as self-respondents at the final follow-up interview.

### Health lifestyles

We included four baseline measures of the healthy aspects of the participant's lifestyles. The first was engaging in vigorous physical activity. Participants were asked "On average over the last 12 months have you participated in vigorous physical activity or exercise three times a week or more?" A binary marker contrasted affirmative with negative responses. Body mass was measured by height to weight ratios, with two binary markers contrasting obese (BMI ≥ 30) and underweight (BMI ≤ 18) participants with all others [22]. Cigarette smoking was measured by two binary markers contrasting self-reported current and former smoking with the reference category of never having smoked. Alcohol consumption was measured by contrasting self-reports of averaging one or more alcoholic drinks per day.

### Continuity of care

We used a previously validated measure of continuity with a primary care physician [23,24]. First, we calculated the percentage of days between baseline and the final follow-up interview for which continuity of care existed. Continuity was defined as existing if no more than an 8-month interval occurred between office visits to the same primary care physician, based on data taken from the Medicare Part B Outpatient and Carrier SAFs. We then converted this percentage into two binary indicators reflecting the extremes of always or never having continuity of care, with having continuity of care some of the time as the reference group.

### Hospitalizations

Arising from the theory of health capital [25], hospitalizations (taken from the Medicare Part A Inpatient SAF) between baseline and the final follow-up represent unexpected changes in health states that capture sea changes in one's health state sufficiently salient to require institutional care [26]. Following Hadley [27], we used three binary markers to contrast quartiles in the distribution of the number of hospitalizations occurring after baseline but before the last follow-up interview. Given the unequal follow-up lengths (i.e., 2-12 years), we calculated these quartiles relative (normalized) to the length of the follow-up period (in years).

### Terminal drop

Substantial decline in cognitive status has been hypothesized and demonstrated to occur in the last year of life, and is referred to as terminal drop [25-28]. But as noted above, the association of terminal drop with functional status has not been explored. To capture participants who may have experienced terminal drop, we included an additional set of three binary indicators. The first reflected whether each participant's final follow-up interview occurred within one year of their death date listed in the Medicare claims Denominator SAF, and was coded "1" for yes and "0" for no. The second indicator reflected whether each participant's death date occurred at least one year and one day after their final follow-up interview. The binary indicator reflecting participants who survived throughout the observation period was used as the reference category.

### Covariates

Based on the available literature [2-7,13,14], other risk factors for functional decline that have been considered or recommended include demographic and socioeconomic characteristics, disease history and comorbidity, and health status. We adjusted for these factors by including them in our analyses. Demographic and socioeconomic factors included age, sex, race, marital status, education, and income. Disease history was measured by a set of 10 binary indicators for whether the participant had ever been told by a physician that she had the particular diseases, and an additional binary indicator for having 3 or more of the diseases tapping comorbidity (or multiple comorbidity). Health status included standard measures of vision, hearing, depressive symptoms, and cognition [27]. Post-baseline primary care physician visits was summed across the observation period using the Medicare Part B claims Outpatient and Carrier SAFs.

The depressive symptoms (8-item Center for Epidemiologic Studies-Depression [CESD-8] scale) and cognition (7-item Telephone Interview for Cognitive Screening [TICS-7], immediate word recall, and delayed word recall) measures, however, were only obtained for self-respondents. Therefore, because our sample includes proxy-respondents to enhance generalizability, it was necessary to transform these measures into four sets of binary indicators. The CESD-8 was transformed into markers for reporting two or more symptoms, or for not having been asked these items, vs. the reference category of reporting none or only one of the symptoms. The TICS-7, immediate and delayed word recall measures were each transformed into markers for being in the lower half of a median-split, or for not having been asked these items, vs. the reference categories of being in the upper half on the median splits. While this approach handles the missing data problem, it creates multicollinearity with the respondent status marker for being having a proxy-respondent at both the baseline and final follow-up interviews. Accordingly, the depressive symptoms and cognition markers were used in an initial analysis that excluded the redundant marker for having a proxy-respondent at both the baseline and final follow-up interviews. Those analyses (not shown) revealed that the cognitive measures were not independently associated with ADLs, IADLs, or mobility declines, and that only the missing depressive symptoms marker was consistently associated with declines in functional status. Inasmuch as that effect is captured by the respondent status indicator for having a proxy-respondent at both the baseline and final follow-up interviews when it is in the model, the depressive symptoms and cognition variables were dropped from all subsequent analyses.

### Adjusting for managed care status

To capture the additive effect of managed care status, a binary indicator was created. It was coded "1" if at any time during the observation period the respondent was enrolled in a Medicare managed care plan, and "0" otherwise. Because only Medicare Part B claims reporting requirements are somewhat relaxed for managed care plans, the only variables for which the interaction of the managed care status indicator is appropriate are continuity of care and the volume (quartiles) of primary care physician visits between the baseline and final follow-up interview. Therefore, simple multiplicative interaction terms were calculated for having been in managed care with the non-reference binary indicators for continuity of care and primary care physician visits. Because none of these multiplicative interaction terms were ever statistically significant predictors of declines in functional status, they were not included in the final models reported here.

### Analysis

The three binary dependent variables were the onset of two or more difficulties (or inabilities) in performing ADLs, IADLs, and mobility tasks. Separate multivariable logistic regression models were estimated to evaluate the effects of the focal and other risk factors on each dependent variable. We used the C-statistic to assess the overall fit (area under the curve) of each model [28]. Statistical assumptions about the logistic regression models were evaluated using standard procedures described by Harrell et al. [29].

### Human subject approvals

The research reported here was supported by a National Institutes of Health (NIH) grant (R01 AG022913) to Dr Wolinsky. The research and restricted data protection plans associated with this grant were approved by AHEAD on February 20, 2003 (#2003-006). Furthermore, the human subject protocol for this study was fully approved by University of Iowa Institutional Review Board (IRB-01) on March 24, 2003, and was fully approved again by IRB-01 at each of its annual reviews. A formal Data Use Agreement with the United States CMS (DUA 14807) for this study was fully approved on March 3, 2005. Written informed consent was obtained from all of the AHEAD participants.

## Results

### Baseline sample characteristics

At baseline, the 5,871 AHEAD participants in the analytic sample were relatively healthy, with only 0.3 ADLs, 0.3 IADLs, and 1.2 mobility limitations on average. The mean age was 77, 62% were women, 9% were African-Americans and 4% were Hispanic. About half were currently married and nearly 40% were widowed. Educational attainment was varied, with 24% having only attended grade school and 28% having gone to college. One-third had 3 or more chronic conditions, with hypertension (44%) and arthritis (24%) being the most common. Three-fourths reported having at least good vision and hearing. Among the self-respondents at baseline (note that proxy-respondents were not asked these questions), the mean number of depressive symptoms was 1.5 (out of 8 possible; *SD *= 1.9), and the average score on the TICS-7 [27] was good at 12.3 (out of 15 possible).

### Focal variables

At baseline, 31% of the analytic sample engaged in vigorous exercise at least three times a week. Obesity was indicated for 14%, while only 3% were classified as underweight. Nine percent were current smokers, while 42% were former smokers. Twelve percent reported consuming one or more alcoholic drinks daily. Twenty-four percent experienced no hospitalizations between their baseline and final self-reported follow-up. Among those with one or more hospitalizations between baseline and the final follow-up, the three remaining quartiles reflected 0.01 to 0.23, 0.24 to 0.50, and 0.51 or more hospitalizations per year of follow-up. Six percent of the participants had post-baseline continuity of care all of the time, while 28% never had continuity. Terminal drop was indicated for 1,778 participants (30.3%), and 37.1% died more than one year and one day after their final follow-up interview, with 32.6% surviving to the end of the observation period. Seventy-two percent were self-respondents at both baseline and the final follow-up interviews, 21% were self-respondents at baseline but not at the final follow-up, 2% used proxy-respondents at baseline but were self-respondents at the final follow-up, and proxy-respondents were used at both interviews for 5%. Nearly 1-in-4 participants (23.3%) were in Medicare managed care at some point during the observation period.

### Follow-up length

The average length of time between baseline and the last self-respondent re-interview was 8.0 years. The final follow-up interview occurred for 913 participants (15.6%) in 1995, 868 (14.8%) in 1998, 816 (13.9%) in 2000, 691 (11.8%) in 2002, 894 (15.2%) in 2004, and 1,689 (28.8%) in 2006.

### Descriptive data for the outcome measures

Overall, 36.6% developed at least two new ADL limitations, 32.3% developed at least two new IADL limitations, and 30.7% developed at least two new mobility limitations. Although some meaningful improvement in function also occurred, it was very modest at 1.6%, 1.3%, and 7.7%, respectively. To determine whether there were differential pattern mixtures involving the extent of functional status change over time for each of the three outcome measures, we conducted stratified analyses based on the year of the final self-reported follow-up (not shown). The general pattern was one of monotonic increases in the percentage with declining health for each of the three outcome measures as the length of the follow-up period increased, as well as monotonic increases in the amount of decline. That is, the longer the exposure period (i.e., the more aging that occurred for the participants), the greater the prevalence of decline and the greater the magnitude of that decline. Thus, state change models (i.e., multivariable logistic regression) reflecting the functional decline from the baseline to the last follow-up interview are appropriate, because there were no differential pattern mixtures over time.

### Multivariable logistic regression models

Columns 1-3 in Table 1 contain the results for the ADL, IADL, and mobility outcomes. Although all covariates were included in these analyses, only the focal variables are shown in the tables for simplicity. Complete tables showing the effects of all of the adjustment variables (covariates) in the model are available on request. Overall, the C-statistics indicate good fits (generally considered to be ≥ 0.70) for all three models, ranging from 0.77 to 0.85.

**Table 1:** Adjusted odds ratios from the multivariable logistic regression models predicting meaningful declines in ADL, IADL, and mobility abilities among the 5,656 Survey on Assets and Health Dynamics among the Oldest Old (AHEAD) participants with complete data

Independent Variables	Decline in ADL Abilities	Decline in IADL Abilities	Decline in Mobility Abilities
*Baseline Function*			

ADL Count	0.782***		

IADL Count		0.690***	

Mobility Count			0.370***

*Exposure Adjustments*			

Centered Years Between Interviews	1.194***	1.224***	1.149***

Centered Years Between Interviews Squared	1.013***	1.008*	0.995

*Respondent Status Adjustments*			

T_1_Self--T_2_Proxy^a^	6.101***	18.620***	4.151***

T_1_Proxy--T_2_Self^a^	0.170***	0.512	0.637

T_1_Proxy--T_2_Proxy^a^	4.343***	7.165***	2.743***

*Respondent Status with Baseline Function Interaction Terms*			

Baseline Function with T_1_Self--T_2_Proxy	0.850	0.570***	0.721***

Baseline Function with T_1_Proxy--T_2_Self	1.372	0.979	0.746

Baseline Function with T_1_Proxy--T_2_Proxy	0.772*	0.687**	0.736*

*Health Lifestyles*			

Engages in Vigorous Activity	0.767***	0.637***	0.634***

Obese^b^	0.989	1.128	1.441***

Underweight^b^	0.940	1.061	0.820

Current Cigarette Smoker^c^	0.788	1.185	1.792***

Former Cigarette Smoker^c^	0.951	1.077	1.059

≤ 1 Alcoholic Drink Daily	0.897	0.839	0.778*

*Continuity of Care *			

Daily After Baseline^d^	0.819	0.837	0.891

Never After Baseline^d^	1.017	1.023	1.058

*Post-Baseline Hospitalizations*			

None^e^	0.481***	0.362***	0.391***

0.01 to 0.23 Annually^e^	0.581***	0.465***	0.545***

0.24 to 0.50 Annually^e^	0.669***	0.708***	0.867

*Managed Care Status*			

Ever In Managed Care	0.975	1.131	1.165

*Terminal Drop*			

Died within 1 Year^f^	1.698***	3.105***	1.635***

Died after 1 Year^f^	1.221	2.475***	1.214

*Model Fit Statistics*			

Area Under the Curve (C statistic)	0.767	0.849	0.817

Adjusted for age, sex, race, marital status, education, income, a set of 10 binary indicators for whether the participant had ever been told by a physician that she had particular diseases, a binary indicator of 3 or more of the diseases tapping comorbidity, vision, hearing, and post-baseline primary care physician visits.

* = p < 0.05; ** = p < 0.01; *** = p < 0.001

^a^Reference category is T_1_Self--T_2_Self.

^b^Reference category is normal weight or overweight.

^c^Reference category is never having smoked cigarettes.

^d^Reference category is continuity of care for 1-99% of days post-baseline.

^e^Reference category is ≥ 0.51 annual hospital episodes.

^f^Reference category is surviving to the end of the follow-up period.

For all three functional decline outcomes, the statistically significant *AORs *for the baseline values in the ADL (0.78; column 1), IADL (0.69; column 2), and mobility (0.37; column 3) models indicated a floor effect such that those with poorer function could not decline as easily as those in better function at baseline. The *AORs *for the exposure time adjustments (i.e., aging effects) indicate that the longer the exposure period (i.e., the more the participants aged) between the baseline and last follow-up assessment, the greater the likelihood of functional decline. There are significant linear exposure/aging effects for all functional outcomes, plus significant non-linear effects for ADLs and IADLs. The non-linear effects indicate that the aging effects get stronger as the participants get older.

The *AORs *for the respondent status markers reflect the additive differences between the four possible groups. As expected, these *AORs *indicate that participants who transitioned from self-respondent status to proxy-respondent status had the greatest risk of functional decline (compared to the reference group of self-respondents at both interviews). Also as expected, participants for whom proxy-respondents were used at baseline but who subsequently became self-respondents have the same or even less risk for functional decline than participants who were self-respondents at both interviews. Participants for whom proxy-respondents were used at both interviews had increased risk of functional decline, although that risk appears to be somewhat less than that for participants who started out as self-respondents but subsequently had to rely on proxy-respondents.

The *AORs *for the interaction terms reflect the differential effects of the baseline functional status measures on functional decline across the four possible respondent status groups. These results are also consistent with our expectations. That is, the *AORs *for the interaction terms involving participants for whom proxy-respondents were used at the final follow-up interview indicated reductions in the risks of functional decline associated with the baseline functional status measures. And, the effects of the baseline functional status measures on functional decline for participants who were self-respondents at both interviews and for those proxy-respondents at baseline who subsequently became self-respondents at the follow-up interview were not significantly different.

Engaging in vigorous activity significantly reduced the odds of functional decline for all outcomes by 23-36%. Body mass, cigarette smoking, and alcohol consumption were significantly associated only with mobility declines. Obesity was associated with a 44% greater odds and being a current smoker was associated with 79% greater odds of mobility decline. Alcohol consumption reduced the odds or mobility decline by 22%. Continuity of care with a primary care physician was not independently associated with declines in ADL, IADL, or mobility function.

Not having any post-baseline hospitalizations prior to the final follow-up interview significantly reduced the odds of declines for all three outcomes. When compared to those experiencing the most post-baseline hospitalizations, the odds of functional decline were reduced by 29-63%. As the average annual number of post-baseline hospitalizations increased (i.e., the first and second tertiles of prior hospitalizations), the odds reduction diminished in a consistently monotonic pattern.

Finally, terminal drop was significantly associated with declines on all three outcomes. Participants who died within one-year of their final follow-up interviews had 54-211% increases in the odds of functional decline, compared to survivors. Among decedents not in the terminal drop window (i.e., those whose death was more than one year and one day after their final follow-up interview), the odds of functional decline was significantly greater only in terms of IADLs (148%).

## Discussion

Our findings fall into two groups: methodological and substantive. The methodological findings involve the significant and substantial additive and interactive effects of respondent status (self- vs. proxy-respondent), and the absence of any effects associated with participating in Medicare managed care. The importance of patient-reported outcomes in health services research, comparative effectiveness research, and health care quality assessment is well-recognized and plays a signal role in health care reform in the United States and elsewhere. For example, one of the major regulatory components of the PPACA legislation [12] was the establishment of the Patient-Centred Outcomes Research Institute (PCORI) under Subtitle D [http://www.pcori.org/]. PCORI's mission is to "is to support the production of well-validated scientific evidence to assist the nation in making informed decisions about a broad range of health care-related issues" [30]. At the centre of this mission is PCORI's guiding principle [http://www.pcori.org/aboutus.html] that states:

"...the patient's voice should be heard in the health care decision making process. PCORI's research will be responsive to the preferences, values and experiences of patients in making health care decisions and the impact diseases and conditions can have on daily life."

That said, it is worth noting that the PPACA statute itself [12] does not define patient-centred outcomes research, and that PCORI's Methodology Committee has proposed no less than four competing definitions [http://pcori.org/images/MC_Report_03-08-2011.pdf] for consideration at the July 2011 PCORI meeting [31].

But however PCORI, or anyone else for that matter, chooses to define patient-centred outcomes research it will be fundamentally driven by patient reports and preferences from a population-based perspective and that most likely will involve longitudinal designs to evaluate the efficacy and effectiveness of competing treatment regimens. This dictates a focus on population-based patient surveys, which inevitably will result in the use of proxy-respondents, especially for older adults [8,9] as the Medicare population may be viewed as an initial target of opportunity for PCORI because of its uniform claims format. Therefore, it is imperative to understand if and how proxy-reports differ from self-reports, and whether those differences affect the results of studies of changes in patient-centred outcomes.

Our research focused on three of the most salient patient-reported outcomes for older adults--ADLs, IADLs, and mobility. We found that not only were there significant and substantial additive effects of respondent status on declines in these standard functional status assessments, which can be rather easily captured by incorporating a set of indicator variables for respondent status, but that respondent status also interacted with (or moderated) the effect of baseline functional status on functional status declines. Generally speaking, there are four plausible explanations for such interaction findings [9:844]:

"(1) beneficiary differences in observed characteristics related to survey response or difficulty to treat..., (2) beneficiary differences in unobserved characteristics..., (3) proxies might report differently on the same experience than an unassisted beneficiary would..., and (4) proxies might report beneficiary characteristics...differently than the beneficiaries would."

Consistent with the approach of Elliott and colleagues [9:844], we interpret the respondent status interaction effects that were observed in the present study as "nonexperimental estimates of the effect of proxy respondents" that otherwise (if only simpler additive adjustments were made) would have resulted in biased parameter estimates when modeling functional status decline.

Moreover, in this context it is important to note that the three standard measures of function status decline examined in our study (ADLs, IADLs, and mobility) represent some of the more "objective" patient-reported outcomes. They are considered to be more objective because ADLs, IADLs, and mobility reflect the target individual's ability to perform observable tasks. As such, our findings for the additive and interactive effects of respondent status on patient-report outcomes likely represent just the tip of the iceberg. As others have suggested [8,9], the more "subjective" the patient-reported outcome is, like perceived health, general well-being, patient satisfaction, or health care quality, the greater the likelihood that the additive and interactive effects of respondent status will be substantially larger.

Therefore, it would seem reasonably straightforward that further research on the additive and interactive effects of respondent status in longitudinal studies of treatment efficacy and effectiveness that rely on patient-reported outcomes is warranted. In addition to exploring whether the magnitude of proxy-respondent bias is inversely associated with the objectivity-subjectivity dimension of the measures under consideration, other aspects of respondent status should also be considered. In particular, bringing information on the reason for the use of a proxy-respondent and the relationship of the proxy-respondent to the target individual to bear on such research is crucial. For example, our results suggest that there are significant differences in estimation bias based on whether the target individual was unavailable vs. unable to answer on her or his own. And, it is likely that proxies having more opportunities to observe the target individual on a daily basis, like spouses, would introduce less estimation bias than proxies who observe the target individual less often, like visiting sons or daughters.

The other methodological finding that warrants mention is the absence of any effect of participating in Medicare managed care plans on the estimation process. Because claims-reporting requirements for Medicare managed care plans are somewhat different for Part B (outpatient services) than they are for fee-for-service Medicare [11], studies using Medicare claims generally have excluded participants who participate in Medicare managed care during any part of the study's observation period. This approach significantly limits their generalizability, especially for studies with longer observation periods. On the one hand, in the absence of prior studies that have investigated this potential bias, our results suggest that such exclusions might be unnecessary. On the other hand, further research is needed to determine whether similar null findings would accrue from studies in which the outcomes being observed were more dependent on the veracity of the Part B Medicare claims for participants in Medicare managed care plans. This is especially important when some aspect of the treatment regimens under study are measured using information from Medicare's Part B claims files.

In the substantive grouping, four of our findings warrant discussion. These involve health shocks, terminal drop, health lifestyles, and continuity of care. The first involves the associations with hospitalizations occurring after the baseline interview but before the final follow-up interview. Based on the theory of health capital [25], these hospitalizations reflect unexpected changes in health states that were sufficient to require institutionalization [26]. We found that post-baseline hospitalization was the most robust predictor of functional decline. Participants who did not experience any post-baseline hospitalizations had statistically and substantively significantly reduced odds of functional decline on all three outcome measures, with those odds reductions ranging from at least 29-63% compared to those who experienced the most post-baseline hospitalizations. Moreover, without exception, there was a dose-response pattern of increased risk for functional decline as the number of post-baseline hospitalizations increased. This confirms the findings related to ADL declines over the short-term (two-years) that were reported from two smaller community-based studies [13,14], and extends those results to include IADLs and mobility in a large sample of Medicare beneficiaries nationally representative of the United States over 2-12 years. Although policy interventions to reduce the number of post-baseline hospitalizations may be difficult to design and implement, our findings suggest the potential for considerable benefit. Moreover, our findings also indicate that there was considerable omitted variable bias in nearly all extant studies that have not considered the role of prior hospitalization.

Among extant interventions to reduce hospitalization risk, post-acute care transition planning and coordination are the most promising [32-38]. A recent review of 21 randomised controlled studies evaluating transitional care planning interventions found that nine provided clear evidence of reduced re-hospitalization rates [39]. This is especially important because reducing re-hospitalization rates is the primary focus for both Medicare and *PPACA *policies directed towards reducing costs and improving care quality [12]. The two common core elements of these nine successful interventions involved assigning a nurse clinical manager to coordinate both the inpatient and post-discharge care, and providing in-home visits after discharge [39]. Medicare is now soliciting demonstration projects that would employ these interventions on a broader scale as part of its Community-Based Care Transitions Program under Section 3026 of *PPACA *[12].

The second finding deserving discussion is the role of terminal drop in functional decline [15-18]. We captured terminal drop with a marker of whether the participant died within one year of their final follow-up interview. Adjusting for all other factors, we found that participants whose final follow-up interview occurred in the terminal drop period had 70% greater odds of decline in ADL abilities, 210% greater odds of decline in IADL abilities, and 64% greater odds of decline in mobility abilities compared to survivors, and significantly greater odds of functional decline that participants who lived more than one year beyond their final interview before dying. To our knowledge, this is the first report that evaluates the role of terminal drop in functional decline among older adults.

The policy implications of these terminal drop findings, however, are not straightforward. Basically, this is because of the fundamental difficulty in prospectively identifying individuals at the end-of-life. Currently, to be eligible for hospice care Medicare requires two independent physicians to certify that the target individual has six months or less to live. While the mean length of hospice care treatment for Medicare beneficiaries is currently 67 days, it clearly has been on the rise, with a substantial number of beneficiaries receiving hospice care well beyond the expected six-month period. In the United States, the National Institute for Nursing Research (NINR) has taken a leadership role on this issue, and has scheduled a major conference [http://www.ninr.nih.gov/ResearchAndFunding/scienceofcompassion] on end-of-life care for August 2011. According to the NINR, going forward any research agenda should focus on: better end-of-life planning, especially for those who are vulnerable and unable to express their preferences; identification of factors that improve strategic decision-making at the end-of-life; improving palliative care and enhancing the lives of dying patients; and, increased interdisciplinary training of a new generation of end-of-life researchers.

The third finding deserving discussion is the role played by lifestyle. We found that engaging in vigorous physical activity reduced the odds of functional decline for all three outcomes from 23% to 37%. Furthermore, obesity and cigarette smoking increased the odds of functional decline in mobility abilities by 44% and 79%, respectively. Because appropriate intervention methods are reasonably well established in the literature for increasing exercise, reducing weight to height ratios, and smoking cessation in both the United States and elsewhere, these findings suggest that the most effective approach to promoting functional health and preventing disability among older Americans lies in the realm of public health policy [1]. This is especially salient given the current focus in the United States on the patient-centered medical home (PCMH) [40,41], inasmuch as PCMHs emphasize prevention, early identification and management of health problems, and quality of life. Moreover, focusing on interventions that would increase exercise, reduce weight to height ratios, and increase smoking cessation is also consistent with the longstanding position of the United States' Institute of Medicine [42] as well as *PPACA *[12].

The final finding to discuss is the absence of any association between continuity of care and functional decline. This has potentially important policy implications because a significant feature of the PCMH [40,41] underlying *PPACA *[12] is continuity of care with a primary care provider [42]. Therefore, a protective effect of continuity of care on functional decline was expected. There are at least two reasons why that effect may not have been observed here. The first is that the Medicare claims-based measure of continuity of care [23,24] used in this study was not sufficiently sensitive to provide an accurate assessment of continuity's role in promoting and maintaining functional status. The second is that while continuity of care may enhance care quality, improve self-rated health and general well-being [43], and reduce mortality risk [24], it simply may not be able to protect older adults against normative functional decline. Further research on continuity of care is needed, however, to evaluate these and other possible explanations.

The strengths of our study notwithstanding, it had three important limitations. First, this was an observational study. While we found that several modifiable factors have significant protective associations with functional decline, we have no direct evidence from this study that increasing vigorous exercise, eliminating obesity, or stopping cigarette smoking would prevent functional decline in these Medicare beneficiaries. In terms of cigarette smoking, however, the evidence is a bit stronger in that we found that there was no additional risk of functional decline for former smokers, suggesting that smoking cessation would improve (by not increasing the risk of decline) mobility. Second, our outcome and risk factor measures were limited to self- and proxy-reports because of the reliance of the AHEAD on telephone interviews. Thus, we were not able to adjust the effects of the focal risk factors for objective measures of physical capabilities like gait speed, grip strength, or balance.

Finally, the separation of aging from period effects is always a challenging task because both are reflected in the length of follow-up (exposure time) measure, and there is no ready solution to the problem [44-49]. That is, in any study design (like ours) that involves people of different ages being followed-up for different lengths of time, exposure time measures capture the potential for both aging and period effects. The best approach is to proceed from strong conceptual grounds, as we have done, by making the assumption that period effects on changes in functional status over this relatively brief time window are highly unlikely [46,47]. Although this assumption is logical and plausible in this case, not even this approach can resolve the underlying and seemingly intractable identification problem [48,49].

## Conclusions

In this study we prospectively examined the risk of long-term functional declines in older Medicare beneficiaries using data on a nationally representative cohort of older American men and women followed for an average of 8.0 years. Several standard measures of functional status were used, and adjustments were made for differential exposure time, respondent status, participating in Medicare managed care plans, a comprehensive set of risk factors, continuity of care, and the influence of post-baseline hospitalizations. We found that (a) the prevalence of decline in functional status was rather common being 36.6% for ADLs, 32.3% for IADLs, and 30.7% for mobility, (b) participants in the terminal drop period had substantial declines in functional status, (c) proxy-respondents were more likely to experience functional decline and proxies had less granular abilities to detect functional status changes, (d) post-baseline hospitalizations induced substantial declines in functional status, (e) aging increased the risk of functional decline with the magnitude of that increase getting larger over time, and (f) good health habits (vigorous exercise) protected against functional decline while bad health habits (obesity and cigarette smoking) increased the risk of functional status decline.

## Competing interests

The authors declare that they have no competing interests.

## Authors' contributions

FDW conceived of the study, wrote the grant application, designed and conducted the analyses, interpreted the results, and prepared the manuscript. SEB, PAMW, and MO performed all of the data cleaning and linkage tasks. MPJ, BK, and JH assisted in the design and oversight of the statistical analyses and their interpretation. RBW participated in the conceptualization of the grant application and the overall study design, and provided clinical expertise. All authors participated in numerous meetings to outline, read, critique, revise, re-read, and grant approval to the final manuscript. FDW and SEB had full access to all of the data in the study and take responsibility for the integrity of the data and the accuracy of the data analysis. All authors read and approved the final manuscript.

## Pre-publication history

The pre-publication history for this paper can be accessed here:

http://www.biomedcentral.com/1471-2318/11/43/prepub
